# Rumen-protected zinc–methionine dietary inclusion alters dairy cow performances, and oxidative and inflammatory status under long-term environmental heat stress

**DOI:** 10.3389/fvets.2022.935939

**Published:** 2022-09-12

**Authors:** Mohsen Danesh Mesgaran, Hassan Kargar, Rieke Janssen, Sadjad Danesh Mesgaran, Aghil Ghesmati, Amirmansour Vatankhah

**Affiliations:** ^1^Department of Animal Science, Faculty of Agriculture, Ferdowsi University of Mashhad, Mashhad, Iran; ^2^Kaesler Nutrition GmbH, Cuxhaven, Germany; ^3^Drug Applied Research Center, Tabriz University of Medical Sciences, Tabriz, Iran

**Keywords:** cattle, heat stress, zinc-methionine, milk, oxidative stress, health

## Abstract

Dairy cows are susceptible to heat stress due to the levels of milk production and feed intake. Dietary supplemental amino acids, particularly rate-limiting amino acids, for example, methionine (Met), may alleviate the potential negative consequences. Zinc (Zn) is beneficial to the immune system and mammary gland development during heat stress. We investigated the impact of a source of a rumen-protected Zn-Met complex (Loprotin, Kaesler Nutrition GmbH, Cuxhaven, Germany) in high-producing Holstein cows during a long-term environmental heat stress period. A total of 62 multiparous lactating Holstein cows were allocated in a completely randomized design to two dietary treatments, namely, basal diet without (control) and basal diet with the supplemental Zn-Met complex (RPZM) at 0.131% of diet DM. Cows in the RPZM group had higher energy-corrected milk (46.71 vs. 52.85 ± 1.72 kg/d for control and RPZM groups, respectively) as well as milk fat and protein concentration (27.28 vs. 32.80 ± 1.82 and 30.13 vs. 31.03 ± 0.25 g/kg for control and RPZM groups, respectively). The Zn-Met complex supplemented cows had lower haptoglobin and IL-1B concentration than the control (267 vs. 240 ± 10.53 mcg/mL and 76.8 vs. 60.0 ± 3.4 ng/L for control and RPZM groups, respectively). RPZM supplementation resulted in better oxidative status, indicated by higher total antioxidant status and lower malondialdehyde concentrations (0.62 vs. 0.68 ± 0.02 mmol/L and 2.01 vs. 1.76 ± 0.15 nmol/L for control and RPZM groups, respectively). Overall, the results from this study showed that RPZM dietary inclusion could maintain milk production and milk composition of animals during periods of heat stress. Enhanced performance of animals upon Zn-Met complex supplementation could be partly due to improved oxidative and immune status.

## Introduction

The evident rise in anthropogenic greenhouse gas emissions in the past decades has resulted in changes in environmental conditions, i.e., more periods of heat stress, challenging livestock, and thermoregulatory mechanism (1). In recent years, researchers were able to decipher the mechanism of heat stress and animal responses at transcription levels in order to introduce novel mitigation strategies (2). Dairy cows are susceptible to thermal stress due to their level of milk production and feed intake. Recent studies have demonstrated the impact of persistent heat stress on dairy production in different environments (3). High genetic merit modern dairy cows undergo thermal stress starting at an average temperature–humidity index (THI) of 68, whereas at a THI of > 72, significant losses in milk production have been observed (4). During environmental heat stress, the amount of energy expended by the cow will increase. The additional energy requirement of the animal must be covered by increasing the dry matter intake (DMI). However, under heat stress, DMI typically decreases, that is, cows will be subjected to unfavorable energy status. Consequently, milk production (both volume and components) and reproductive indices would be impaired (4). A decline in the daily DMI causes lower uptake of nutrients by the animals, particularly amino acids (AAs). Feeding AA-deficient diets has resulted in heat production elevation in the animals, due to the increase in tissue protein turnover (5). Optimizing post-ruminal levels of rate-limiting AAs such as methionine (Met), that is, metabolizable Met concentration, might alleviate the negative consequences of heat stress (5). Including a sufficient amount of AAs in dairy cattle diets has been an important practice in dairy nutritional management. Dietary AAs may enhance health and lactation performance including milk protein and fat yield (6). Methionine is an essential AA for growth and tissue repair and improves the metabolism and health of dairy cows (7). Methionine as a “functional AA” also has important physiologic actions beyond protein synthesis, such as DNA methylation, regulation of translation, and synthesis of other molecules (e.g., choline and polyamines), altering the immune system and oxidative metabolism (7, 8). Recent studies indicated that feeding rumen-protected Met could increase milk protein yield (9). Enhanced milk protein synthesis may be related to the link between Met and branched-chain AAs regarding the regulation of biosynthesis of milk protein (10). Methionine also promotes the absorption of zinc (Zn) (11) and acts as a lipotropic agent to prevent excess fat build-up in the liver (12).

Zinc, one of the most essential trace minerals for animals, is incorporated into a variety of proteins and enzymes that are involved in a wide range of physiological processes (13). Recent evidence has indicated that the organic Zn complex has higher bioavailability than inorganic Zn and hence is widely recommended in monogastrics (14) and ruminants (15). Previous results demonstrated that the dietary supplementation of an organic form of Zn improved immune function (15), antioxidant activity (16), and overall performance (17) in different livestock species. Chen et al. (18) revealed that feed intake and Zn digestibility and deposition in dairy cattle increased linearly with increasing Zn-Met supplementation from 60 days before expected calving until parturition. An organic form of Zn supplementation could enhance milk production and decrease the somatic cell scores (SCC) in lactating cows (19). In addition, dietary organic Zn inclusion would be beneficial to heat shock responses (17), mammary gland development (20), and metabolic responses (21) of dairy cattle during environmental heat stress.

Research on the effect of Zn-Met supplementation in high-producing dairy cows under long-term environmental heat stress (LEHS) is scarce. Therefore, we aimed to observe the impact of a commercially available rumen-protected Zn-Met complex (Loprotin, Kaesler Nutrition GmbH, Cuxhaven, Germany) supplementation in early high-producing Holstein cows during a LEHS period on (a) milk yield and chemical composition; (b) distinct animal behavioral indices such as rumen fill index, manure score, rumination activity, and body condition score (BCS); and (c) plasma concentrations of various metabolic, immune, and oxidative stress biomarkers such as glucose, urea, cholesterol, high-density lipoprotein (HDL), low-density lipoprotein (LDL), triglyceride, and blood enzymes—such as alanine aminotransferase (SGPT) and aspartate aminotransferase (SGOT)—non-esterified fatty acids (NEFA), beta-hydroxybutyrate (BHB), total protein, globulin, albumin, haptoglobin, calcium, Zn, total antioxidant status (TAS), malondialdehyde (MDA), and interleukin-1 beta (IL-1B).

## Materials and methods

### Environmental data

Daily data of ambient temperature (T, °C) and relative humidity (RH, %) throughout July and August 2021 were obtained from Fariman meteorological stations (35° 42′ 0″ N, 59° 50′ 0″, Khorasan Razavi, Iran), located within a 3-km distance from the experimental site. Daily readings were then considered by retrieving the maximum daily T (Tmax) and RH value. The daily maximum THI was calculated, using the equation reported by Kendall et al. (22) and Ouellet et al. (23): THI = (1.8 × Tmax + 32) – [(0.55 – 0.0055 × RH) × (1.8 × Tmax – 26)].

This equation has been proposed for animal experiments conducted in a continental climate condition with large diurnal and seasonal changes in temperature (23). Inserting the daily maximum T in the equation apparently fits better with the data obtained from meteorological stations (23).

### Animals and feeding

The animal study was reviewed and approved by the Institutional Animal Care Committee, Ferdowsi University of Mashhad (Mashhad, Iran; Protocol number 101984). All experimental procedures followed the description of the Iranian Council of Animal Care guidelines (24).

A total of 62 high genetic merit multiparous lactating Holstein cows [684 ± 16 kg of bodyweight, 28 ± 7 days in milk (DIM), parity 2.9 ± 0.6, milk yield 51.8 ± 2.2 kg/d, milk fat 35 ± 0.21 g/L, milk protein 3.23± 0.05 g/L, and BCS 2.76 ± 0.21] were enrolled in this experiment. The cows were balanced by a 305-day previous mature equivalent milk yield, milk yield during the 3rd and 4th weeks of lactation, DIM, and parity, then assigned to two dietary treatments (*n* = 31 per group). The animals were fed the same basal diet for 7 days (5th week of lactation) and then were randomly assigned to two dietary treatments, namely, control (only basal diet) and basal diet plus a rumen-protected Zn-Met complex (RPZM; Loprotin, Kaesler Nutrition GmbH, Cuxhaven, Germany, as 0.131% of diet DM) beginning at the 6th week of lactation for a total period of 6 weeks. The Zn-Met complex source contains 30% DL-Met and 7% Zn. The ingredients and chemical composition of the experimental diets are presented in Supplementary Table S1. During the study, the cows were housed in two separated free stall rows, in a barn of a commercial dairy farm, Moghofat Maleck-Fariman, with 1,120 milking cows. The barn was equipped with four ceiling fans (4 × 3 m, fan/58.5 m^2^), which were working for 24 h/day. The animals had free access to feed and water; the diets were fed as a total mixed ration (TMR) three times per day at 07:30, 16:30, and 22:30 h in amounts that ensured *ad libitum* consumption and ~3–5% feed refusals. Dry matter intake and apparent DM digestibility were determined as proposed by Velásquez et al. (25). An internal marker, that is, naturally occurring indigestible fractions of feedstuff, was used for this method. During the study period, two individuals were constantly monitoring the animals and collecting their feces upon excretion in order to avoid potential contamination with bedding or partial material loss. A marker technique was applied to estimate the fecal output and DM digestibility. Dry matter intake was then calculated by dividing the fecal output by the indigestibility of DM. Samples of the diet ingredients were collected weekly, dried in a forced-air oven for 72 h at 55°C, ground using a Wiley mill to pass a 1-mm screen, and then analyzed for DM and chemical composition (26). Dry matter was determined after 24 h at 95°C (ISO 6496). Ash was determined after 3 h at 550°C (ISO 5984). Nitrogen was assessed using the Kjeldahl method (Kjeltec 2300 Autoanalyser, Foss Tecator AB, Hoganas, Sweden) with crude protein (CP) as N × 6.25. The starch content was evaluated by using an anthrone–sulfuric acid method with glucose as the standard and estimated as 0.9 × glucose content after liberating the starch by heating in a boiling water bath in the presence of 2 N HCl (27). For neutral detergent fiber (NDF) and acid detergent fiber (ADF), the method of Goering and Van Soest (28) was used.

The cows were milked three times daily at ~04:00, 12:00, and 20:00 h. The incidence of health problems was recorded for each cow accordingly throughout the experiment, and appropriate treatment was considered if necessary.

### Sample collection

Feed refusals of each group were measured daily, and the total DMI of each experimental group was monitored by difference assuming a similar DM content of feed offered and the ort. Bi-weekly (weeks 7, 9, and 11 of lactation) samples of rectal feces were obtained from 20 cows in each group to evaluate the fecal output, DM digestibility, and feed intake. Milk yield was recorded daily, and weekly milk samples were obtained at three consecutive milking periods. The samples were preserved with 2-bromo-2-nitropropane-1,3-diol and analyzed for protein, fat, lactose, milk urea nitrogen (MUN), SCC, solids nonfat (SNF), and total solid content by Fourier-transform infrared spectroscopy (FT-IR; CombiScope FTIR 600 HP, Delta instruments, Drachten, The Netherlands) in a commercial laboratory (Sazan Rojan Alvand CO., Iran).

Fat-corrected milk (FCM) standardized to 4% fat was calculated using the following equation: FCM = [0.4 × milk yield (kg)] + [15 × milk fat (kg)], and energy-corrected milk (ECM) was calculated as presented by Muñoz et al. (29): ECM = [0.3246 × milk yield (kg)] + [12.86 × fat yield (kg)] + [7.04 × protein yield (kg)]. The milk energy content (NEL) was calculated using the following equation: NEL = [(0.0929 × % milk fat) + (0.0563 × % milk true protein/0.93) + (0.0395 × % milk lactose)] × milk yield (30).

Blood was sampled bi-weekly (weeks 5, 7, 9, and 11 of lactation) from 15 cows in each group at 08:00 h *via* puncture of the coccygeal vessels using Clot Activator vacutainers (Hebei Xinle Sci&Tech Co., Hebei, China) (31). The samples were kept at room temperature, and the serum was separated within 0.5 h and then frozen and stored at −20°C until analysis for glucose (GOD-PAP, https://parsazmun.ir), triglycerides (GPO-POD, www.Bionik.web.com), NEFA (colorimetric method, RANDOX, www.Randox.com), BHB (kinetic enzymatic method, RANDOX, www.Randox.com), urea (urease-GLDH method, https://parsazmun.ir), cholesterol (CHOD_POG, http://paadco.co), HDL (direct enzymatic colorimetric method, http://paadco.co), SGOT (https://parsazmun.ir), SGPT (https://parsazmun.ir), calcium (https://parsazmun.ir), Zn (colorimetric method, RANDOX, www.Randox.com), total protein (https://parsazmun.ir), albumin (https://parsazmun.ir), haptoglobin (ZellBio GmbH, Lonsee, Germany), TAS (Manual caloric method, RANDOX, www.Randox.com), MDA (thiobarbituric acid reactive substance assay), and IL-1B (ZellBio GmbH, Lonsee, Germany). Both LDL and very low-density lipoprotein (VLDL) were calculated using the following equation: LDL = Total cholesterol – (HDL + VLDL), VLDL = Triglycerides/5.

### Animal behavior and body condition score

Bi-weekly animal behavior indices including the time spent ruminating were recorded every 10 min per 24 h and calculated by multiplying the total number of observed activities in each duration (32). The body condition score, the rumen fill score, and the manure score were assessed bi-weekly from the 7th week of lactation. The body condition score was recorded by the same operator using a 1 to 5 scale with 0.1 intervals, as proposed by Ferguson et al. (33). Both the rumen fill score (34) and the manure score (35) were assessed six times per day starting 2 h before the morning feeding by the same operator using a 1–5 scale. The visual characteristics for scoring the manure and rumen fill of the Holstein dairy cows were carried out, as previously described (34, 35).

### Statistical analysis

In the RPZM group, one cow was removed from the experiment at the end of the 9th week of lactation due to a physical injury. In addition, two cows in the control group were culled at the 7th and 8th weeks of lactation due to a sudden unknown drop in milk production.

Data of milk yield and its component as well as blood biochemistry from the 5th lactation week (i.e., all animals received a similar basal diet) were used as a covariate in the statistical analysis. The main aim of the statistical analysis of the experimental data was to determine the productive and metabolic responses of the cows fed the experimental diets. Data obtained weekly were statistically analyzed using the Proc Mixed procedure of SAS 9.1 (SAS Institute, Cary, NC, USA) for a completely randomized design with repeated measures included by the corresponding covariate. The model included the effects of group, week of lactation, and the interaction between group, week, and covariate (if necessary). The week of lactation was used as repeated measurement, with a cow within experimental groups as the subject. The experimental group, week, interaction between treatment and week, and corresponding covariate were fixed effects, and error was the random effect. Data on DM digestibility were averaged by cow and week of lactation and then analyzed using a completely randomized design. Differences were considered significant at *P* < 0.05, whereas tendency was determined at 0.05 < *P* < 0.1. Data are expressed as mean ± standard error of means.

## Results

### Dry matter intake, milk yield, and milk composition

**Figure 1:**
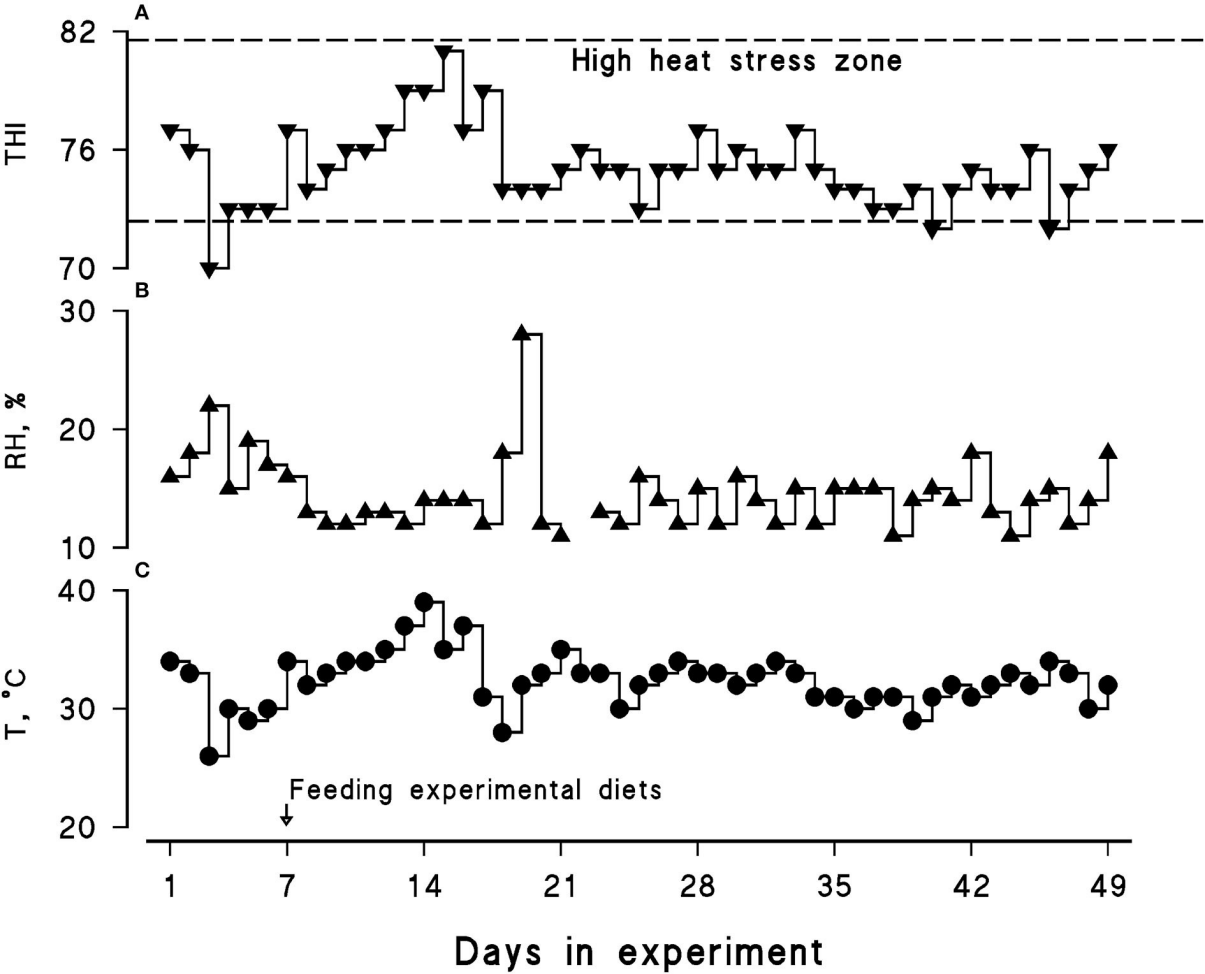
Calculated temperature–humidity index (THI; **A**) along with recorded relative humidity (RH, %; **B**) and maximum ambient temperature (T, °C; **C**) during the course of the experiment (July–August, 2021). The daily maximum THI was calculated using the following equation: THI = (1.8 × Tmax + 32) – [(0.55 – 0.0055 × RH) × (1.8 × Tmax – 26)].

Average ambient temperature, relative humidity, and the THI were 32.5°C, 14.5%, and 75.13, respectively. A daily similar pattern in the THI was observed during the course of the present experiment; hence, all the animals experienced a LEHS as the daily THI was above 70 (Figure 1).

**Figure 2:**
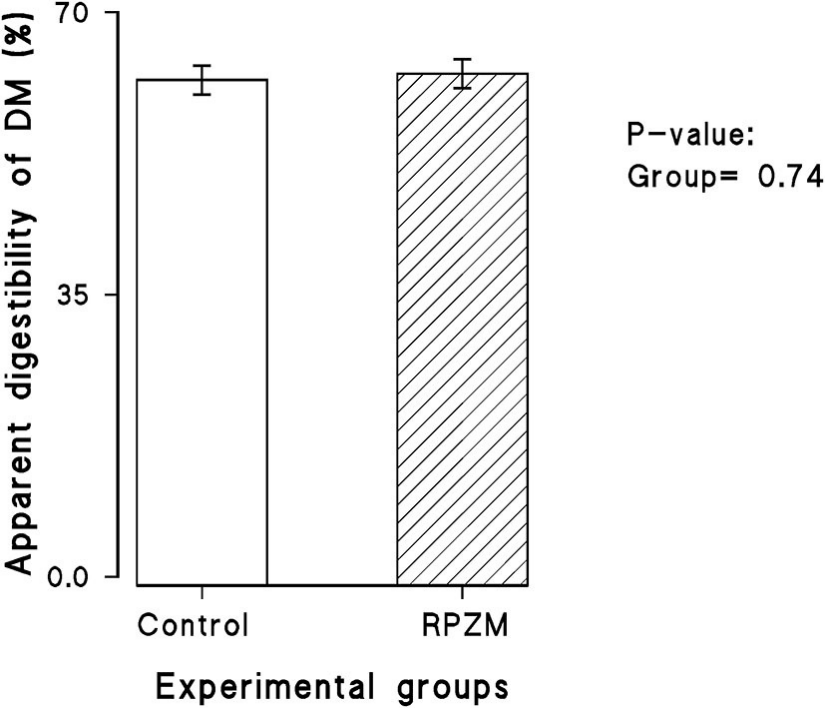
Apparent digestibility of dry matter in high-producing Holstein cows supplemented without (control) or with rumen-protected zinc–methionine complex (RPZM) during long-term environmental heat stress.

The dry matter intake and productive responses of the cows in the experimental groups are depicted in Supplementary Table S2. There was hardly any significant effect in the experimental group and interaction between the week of lactation and the group on the DMI (25.85 vs. 26.68 ± 1.3 kg for the control and RPZM groups, respectively) and apparent total tract DM digestibility (Figure 2). Although milk yield in cows fed the Zn-Met complex was roughly 3 kg higher (52.9 vs. 55.1 ± 1.21 for the control and RPZM groups, respectively), this did not reach the significance level. The cows in the RPZM group had evidently higher milk fat and protein concentration (*P* < 0.05; 27.3 vs. 32.8 ± 1.8 g/ kg and 30.1 vs. 31.0 ± 0.23 g/ kg for the control and RPZM groups, respectively). On the other hand, the milk lactose concentration in the control group was higher than that in the RPZM group (*P* < 0.05; 46.6 vs. 45.9 ± 0.33 g/ kg for the control and RPZM groups, respectively). The milk solid concentration was evidently higher in the RPZM group than in the control (*P* < 0.01; 113 vs. 119 ± 1.75 g/ kg for the control and RPZM groups, respectively), whereas the milk SNF concentration was not affected by the dietary treatment (*P* > 0.05). Milk urea nitrogen in the animals hardly changed upon supplementation with the Zn-Met complex. Yields of fat and protein upon Zn-Met supplementation were on average 25 and 7% higher, respectively, during the course of this experiment (*P* ≤ 0.01). Fat (4%)-corrected milk and ECM were evidently higher (*P* = 0.002 and 0.004, respectively) in the RPZM group than in the control (42.5 vs. 48.8 ± 1.82 kg/d and 46.7 vs. 52.8 ± 1.72 kg/d for the control and RPZM groups, respectively). Moreover, NEL was significantly higher in animals fed the Zn-Met complex source than in the control (*P* = 0.024; 91.1 vs. 100 ± 2.71 Mcal/d for the control and RPZM groups, respectively). The animals in the RPZM group had significantly lower (*P* = 0.001) SCC during the experimental period than their counterparts in the control group.

### Body condition score and animal behavioral indices

**Figure 3:**
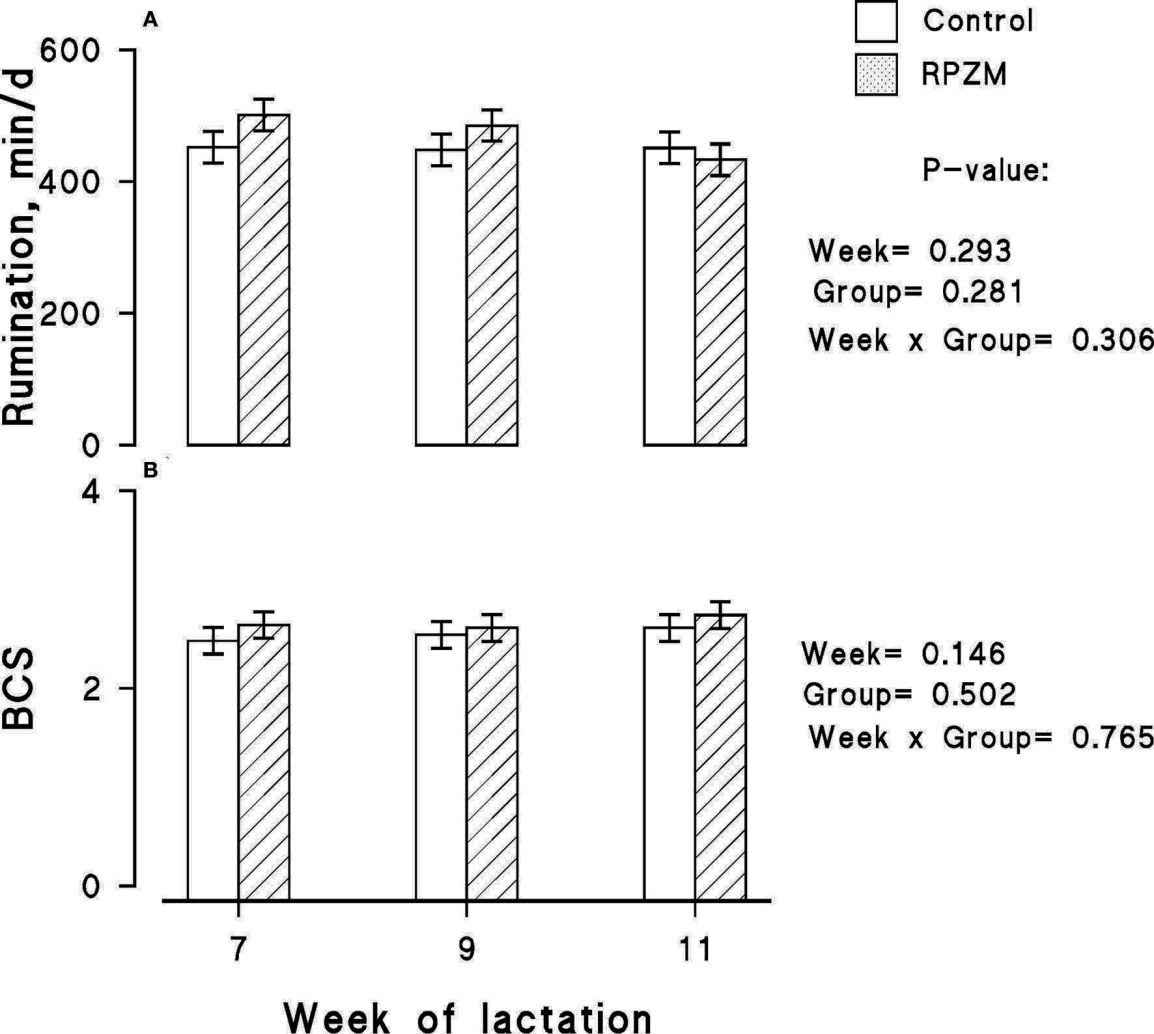
Rumination time **(A)** and body condition score (BCS; **B**) of high-producing Holstein cows supplemented without (control) or with rumen-protected zinc–methionine complex (RPZM) during long-term environmental heat stress. Values are means ± standard error of means.

Data on rumination time per 24 h for each experimental group are presented in Figure 3A, in which no significant differences between the groups and lactation week were observed. The effect of diets on the BCS of the animals throughout the experimental lactation weeks is depicted in Figure 3B. The initial BCS of all animals located in the groups were similar, and hardly a significant effect of the experimental groups was observed in the study.

**Figure 4:**
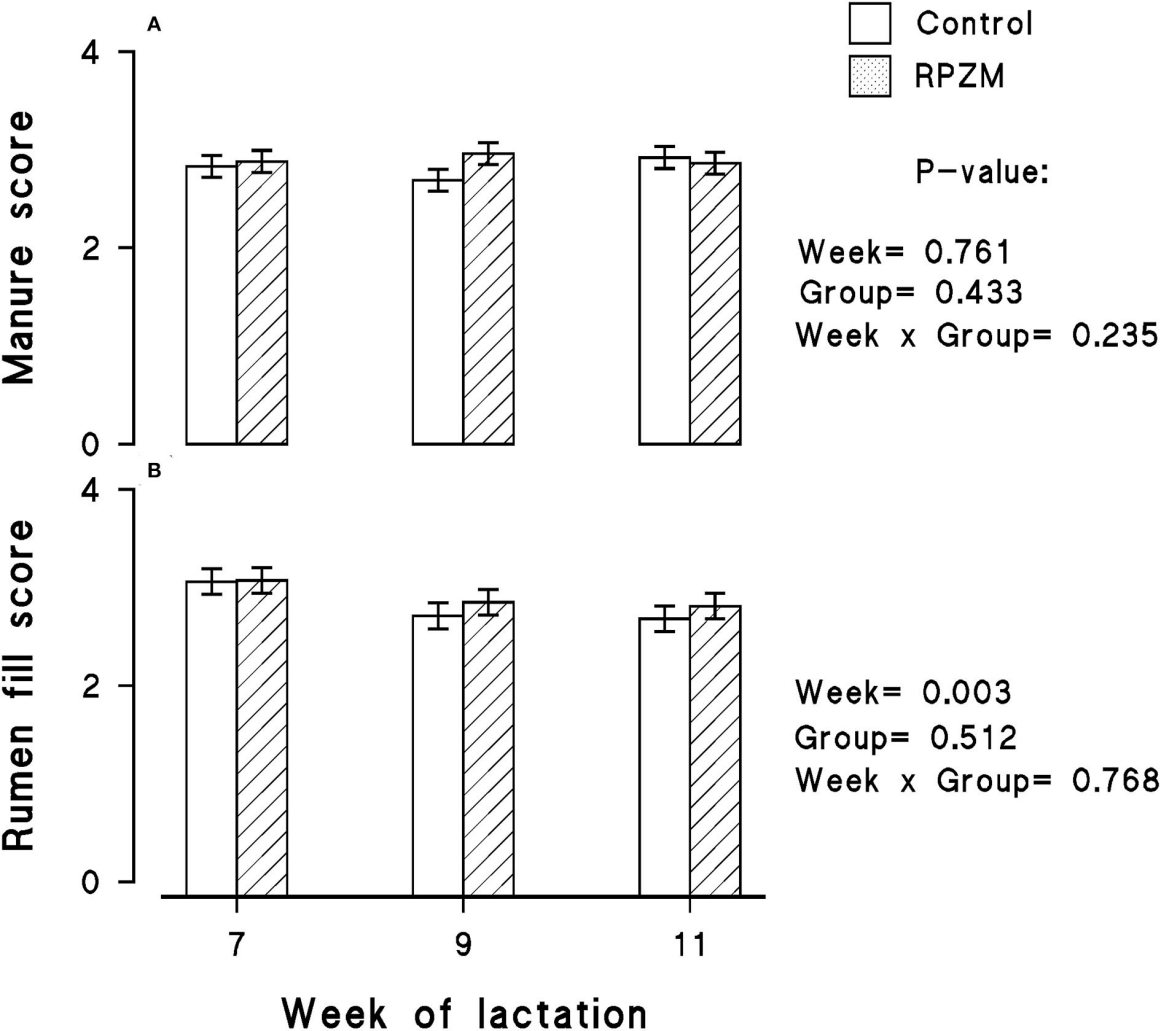
Manure score **(A)** and rumen fill score **(B)** of high-producing Holstein cows supplemented without (control) or with rumen-protected zinc–methionine complex (RPZM) during long-term environmental heat stress. Values are means ± standard error of means.

The data of the manure score and rumen fill score are shown in Figures 4A,B, respectively. The manure score was unaffected by the experimental groups. However, the week of lactation caused a decrease in the rumen fill score within the experimental period (*P* < 0.05). The animals generally had a lower rumen fill score as the experimental lactation weeks progressed.

### Blood serum biomarkers

**Figure 5:**
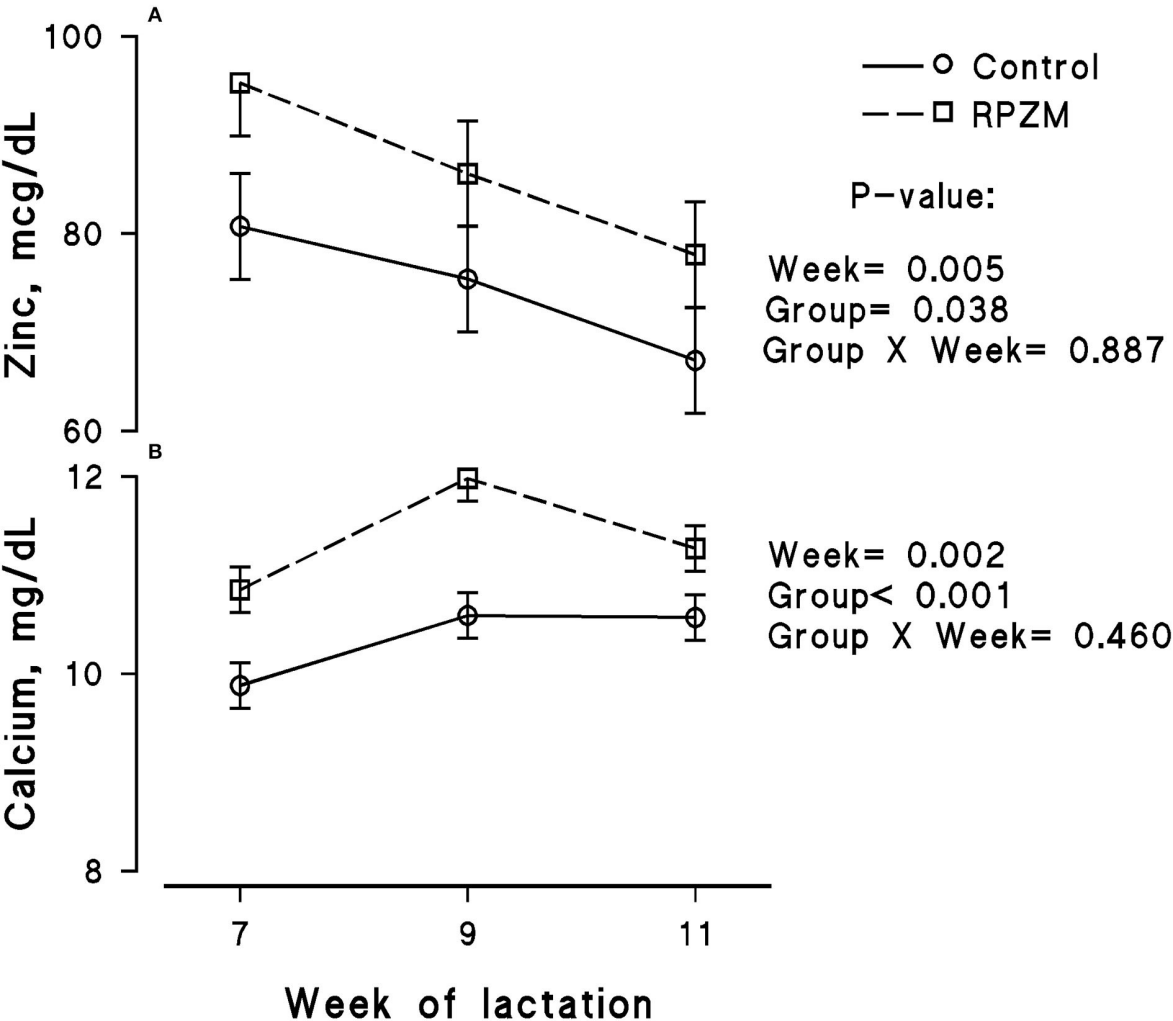
Blood serum concentration of zinc **(A)** and calcium **(B)** of high-producing Holstein cows, through lactation weeks 6–11, supplemented without (control) or with rumen-protected zinc–methionine complex (RPZM) during long-term environmental heat stress. Values are means ± standard error of means.

**Figure 6:**
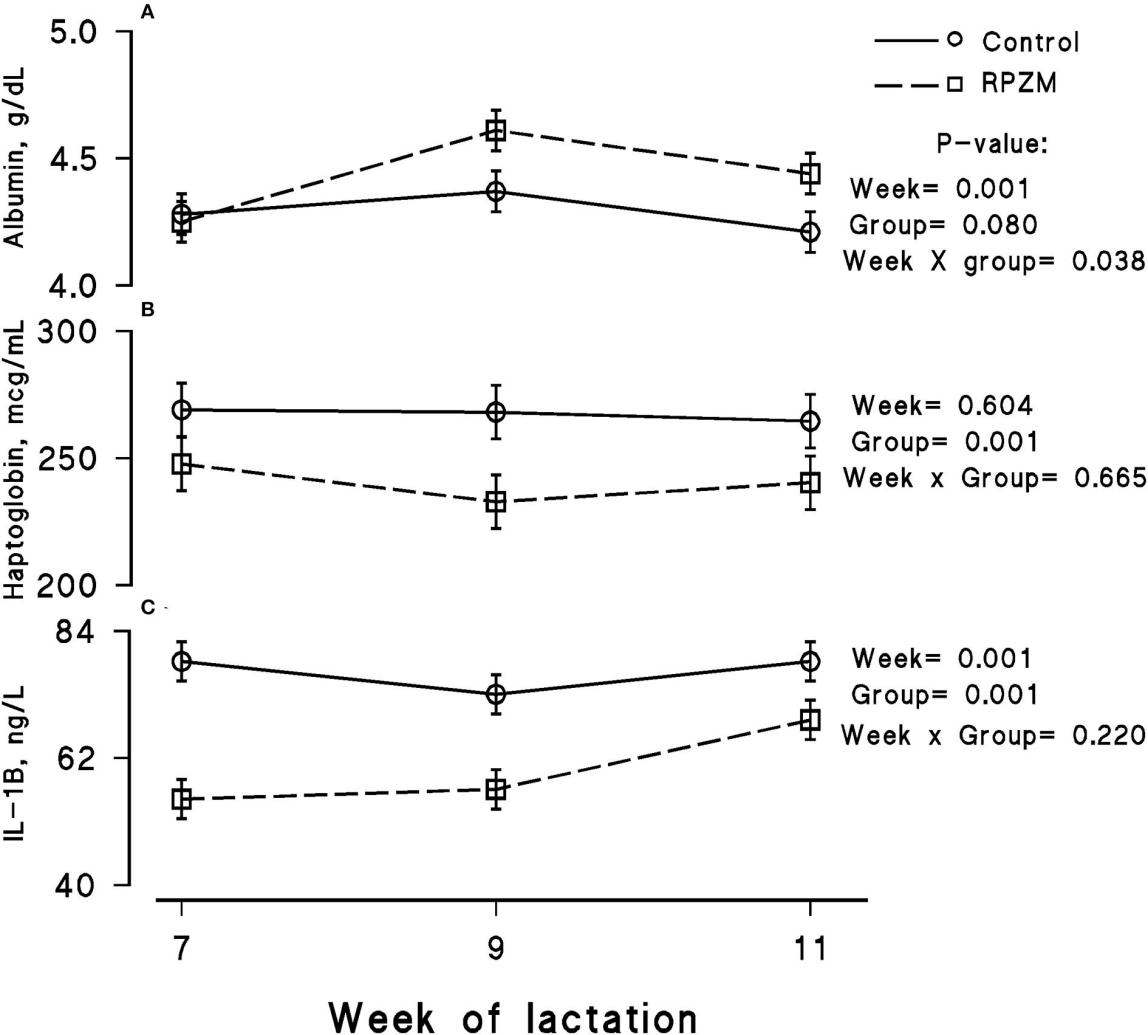
Blood serum concentration of albumin **(A)**, haptoglobin **(B)**, and interleukin-1 beta (IL-1B; **C**) of high-producing Holstein cows, through lactation weeks 6–11, supplemented without (control) or with rumen-protected zinc–methionine complex (RPZM) during long-term environmental heat stress. Values are means ± standard error of means.

**Figure 7:**
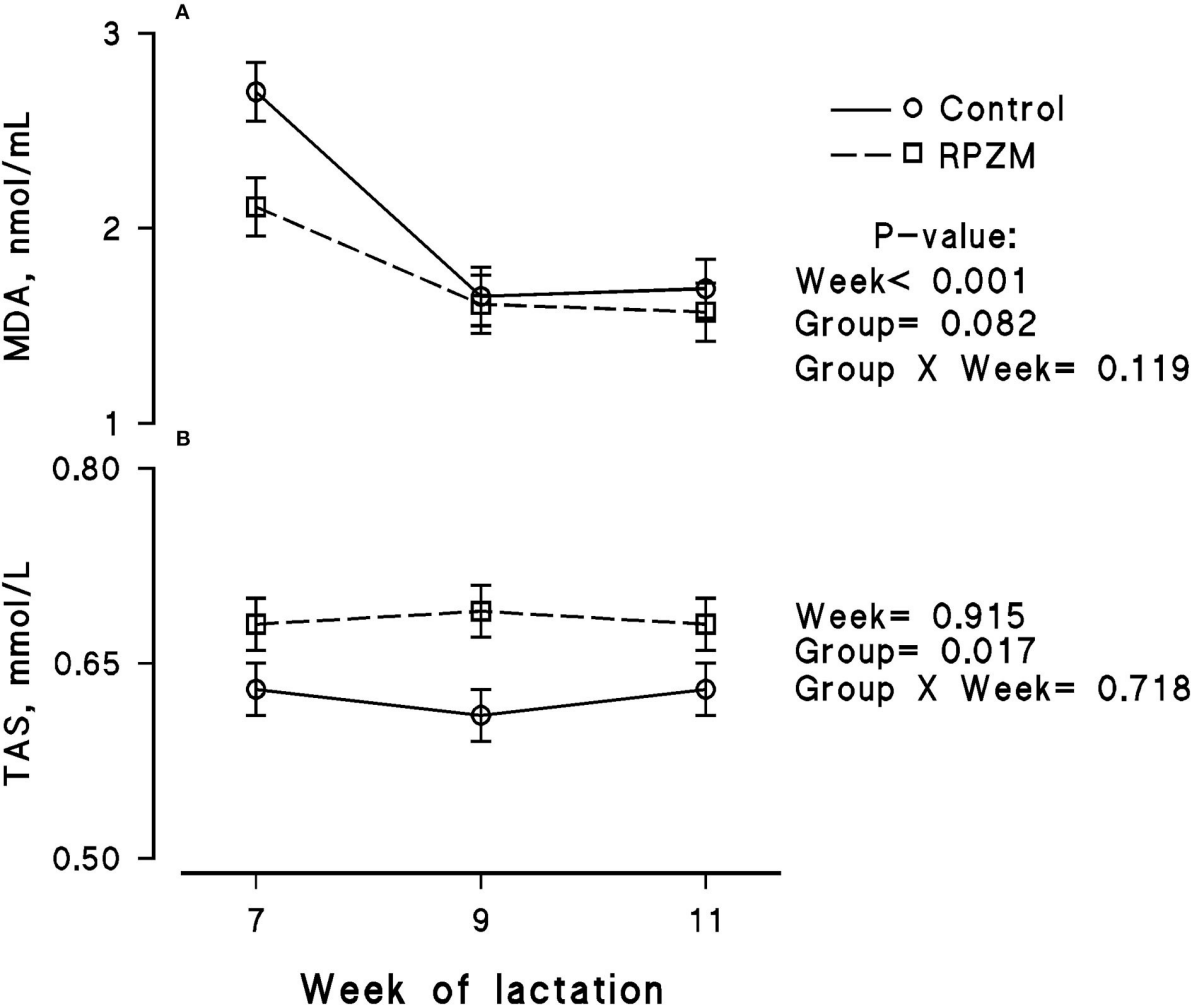
Blood serum concentration of malondialdehyde (MDA; **A**) and total antioxidant status (TAS; **B**) of high-producing Holstein cows, through lactation weeks 6–11, supplemented without (control) or with rumen-protected zinc–methionine complex (RPZM) during long-term environmental heat stress. Values are means ± standard error of means.

The blood serum composition of the cows fed the experimental diets during a LEHS period is shown in Supplementary Table S3. In the current study, the circulating glucose and urea concentrations of the animals between experimental groups were not significantly different. Data regarding the concentration of blood triglycerides, BHB, and NEFA showed hardly any significant difference between the experimental groups (*P* > 0.05). Blood serum cholesterol and LDL concentration showed a tendency to be higher (*P* = 0.1 and *P* = 0.06, respectively) upon dietary supplementation of the Zn-Met complex during the experimental period. However, the circulating HDL and VLDL concentrations were not significantly different between the experimental groups. The circulating concentration of blood enzymes including SGOT and SGPT was not significantly changed by the experimental diet during the course of this study (*P* > 0.05). There was a clear effect of the experimental groups and the week of lactation on blood serum Zn concentration (Figure 5A; *P* = 0.038). The cows fed the basal diet supplemented with the Met-Zn complex source had evidently higher blood serum Zn concentration than those of the control (74.4 vs. 86.4 ug/dL for the control and RPZM groups, respectively). The serum calcium concentration in the animals fed the experimental diets supplemented with the Zn-Met complex was clearly (*P* < 0.01) higher than that in the control (10.3 vs. 11.3 ± 0.23 mg/dL for the control and RPZM groups, respectively; Figure 5B). In this study, the Zn-Met-supplemented cows tended to have a higher albumin concentration than those in the control group (Figure 6A; *P* = 0.08). In addition, the blood serum haptoglobin concentration evidently decreased (*P* = 0.001) in the cows fed the experimental diet with the Zn-Met complex supplementation (267 vs. 240 ± 10.53 mcg/ml for the control and RPZM groups, respectively; Figure 6B). The outcome of this work indicated that the circulating IL-1B concentration in the animals supplemented with the Zn-Met complex was markedly (*P* = 0.001) lower than that in the control group (76.83 vs. 60.01 ± 3.4 ng/L for the control and RPZM groups, respectively; Figure 6C). Blood serum concentrations of MDA and TAS of the animals in both experimental groups are shown in Figures 7A,B, respectively. The dairy cattle in the RPZM group had significant (*P* = 0.017) higher blood TAS along with a lower MDA concentration (*P* = 0.08) throughout the experimental period than their counterparts in the control group (0.62 vs. 0.68 ± 0.02 mmol/L and 2.01 vs. 1.76 ± nmol/L for the control and RPZM groups, respectively).

## Discussion

### Feed intake of the animals

In this study, supplementing the diet of the dairy cows with the Zn-Met complex did not show any effect on the feed intake of the animals. Therefore, it could be postulated that the negative effect of Met on DMI is negligible when a Zn-Met complex is used in the diet of lactating cows under heat-stress conditions. Previous reports showed that an increase in the catabolism of AAs resulted in the elevation of the core body temperature and greater body protein turnover, which is associated with a higher heat increment (36). An increase in deamination of AAs, resulting from an excess intake of the AAs relative to the animal requirements, would contribute to losses in both DMI and production in heat-stressed animals. Robinson et al. (37) infused Met, in excess of the animal requirement, and reported depression of feed intake in the animals. They concluded that the observed reduced DMI might be due to the imbalanced supply of AAs. Kassube et al. (38) revealed that heat stress decreased the DMI by 1.5 kg/d compared with those animals in the thermoneutral condition, while in animals receiving Met and branched-chain AAs, the DMI decreased by 0.7 kg/d.

### Lactation responses of the animals

The outcome of this experiment pinpointed an evident influence of the feeding Zn-Met complex on lactation performance, as evidenced by increased both milk fat and protein concentrations and yields. It is important to focus on the dairy cow diet formulation to cover animal metabolizable Met in order to enhance milk protein and fat production. Recent data showed a positive impact of feeding rumen-protected Met on milk protein concentration (6) and yield (9) of dairy cows exposed to LEHS. However, the influence of rumen-protected Met products in previous research has been inconsistent to some degree, with considerable variations for particular lactation performance responses (39). The outcome of this study was in line with previous work (6) indicating an increase in milk fat yield by enhancing the post-ruminal Met supply. Batistel et al. (7) demonstrated that 0.17 kg/d more milk fat was produced in early lactating Holstein cows upon increasing the metabolizable Met concentration. Moreover, Patton (40) reported that cows with deficient Met responded to rumen-protected Met supplementation with greater milk fat yield. Apparently, Met has the potential to alter the mRNA expression of selected lipogenic genes in bovine mammary cells (41). Nevertheless, the impact of feeding rumen-protected Met on the regulation of mammary fatty acid synthesis merits further investigation. Other plausible reasons for positive effects of feeding rumen-protected Met on milk fat concentration and yield are related to the alleviation of the negative energy balance in dairy cows through improvement in the animal's metabolic status and immune system (7). Kellogg et al. (42) reported that feeding Zn-Met, relative to an inorganic form of Zn, increased the milk composition yield in dairy cattle. Nayeri et al. (43) studied different ratios of zinc sulfate to zinc amino acid complex in pre- and post-partum Holstein cows. They concluded that supplementing organic zinc depicts positive effects on production parameters in dairy cows. The current experiment was able to reveal the positive impact of feeding rumen-protected Zn-Met on FCM, ECM, and NEL in dairy cows exposed to LEHS. This may explain the value of a supplementary mixture of Zn and Met in animals. However, several studies showed an absence of such a positive impact of Zn (17) or Met (9, 38) on FCM and ECM. These non-significant responses could be due to different factors, such as experimental design, number of animals in each treatment, animal homogeneity in each group, and in-group variations among the animals. Recently, the term “residual feed intake” (RFI) has been introduced as a tool to evaluate feed efficiency in dairy cows, as the RFI is independent of the production level (44). The residual feed intake is calculated as the difference between the cow's actual feed intake and its predicted feed intake calculated from milk production, metabolic body weight, and change in body weight (45). Lactating cows with a negative RFI consume less than expected for their level of production and thus are more efficient. The biological mechanisms elaborating differences in RFI among dairy cows are not well-defined (40); however, it has been shown that protein turnover and tissue metabolism have a high impact on RFI in finishing beef steers (46). Hence, in this study, it could be postulated that the positive effect of the supplementary Zn-Met complex on lactation performance may be achieved by optimizing body protein turnover and milk protein generation, leading to a better RFI as the DMI between the groups was similar. On the other hand, current experimental dietary Zn provided by a complex of Zn-Met showed a potent impact in alleviating the negative effects of heat stress on both fat- and energy-corrected milk. Zinc is able to maintain critical body functions including epithelial integrity and tissue function (47). Abuajamieh et al. (48) revealed that dietary inclusion of organic Zn leads to improved intestinal barrier function during heat stress. Environmental heat stress increases the permeability of the mammary epithelium of lactating dairy cows, while dietary Zn helps improve the performance along with maintaining the permeability of the mammary epithelium and mammary epithelial integrity (17). Therefore, AAs and energy may shift to milk synthesis, rather than restoring proper epithelial integrity. Our data confirmed the findings of Sobhanirad et al. (49), which evaluated the effect of a mixture of Zn-Met and an inorganic form of Zn supplement in lactating cow diets and reported lower SCC in the treatment group than in the control. Pate et al. (5) concluded targeting a more balanced AA diet in dairy cattle during heat stress, *via* dietary rumen-protected Met supplementation, maintains lower milk SCC in the animals. The current outcome implies that animals fed the Zn-Met complex could be less prone to experiencing mammary gland disorders, which hinder the productivity and longevity of dairy cows.

### Body condition score and distinct behavioral indices

In this study, neither the experimental diets nor the weeks of lactation showed any evident influence on the animal BCS. Robinson et al. (50) studied the effect of rumen-protected Met on performance and BCS changes in Holstein cows. They concluded that cows supplemented with rumen-protected Met had a similar BCS in relation to other experimental groups. Therefore, most energy intake was used for the output of energy in milk, rather than changes in body weight and BCS. Rumination time is mostly influenced by the physically effective fiber (32), which resulted in increasing the surface area of the feed particle. Furthermore, the time that cows spend chewing might be a valuable management tool for detecting health problems and optimizing the herd health status (32) as monitoring rumination time is easier than monitoring the DMI. In the present study, the mean daily rumination time of the cows located at the experimental site was above the threshold of early lactating Holstein cows (32), indicating that the animals were in good health status. In the current work, the cows had rumen fill scores that are acceptable for high-producing Holstein cows. However, the rumen fill score decreased with the progress of the experimental weeks. The rumen fill score is mainly influenced by feed intake, particularly by the proportion of roughage in the diet (34). Hence, it is believed that the animals used in this study had a sufficient DMI and relatively well-balanced metabolic status.

### Selected biochemical markers

Circulating calcium concentration was higher in the animals fed the commercial Zn-Met source during LEHS. The effect of dietary CP on flux and metabolism of calcium has been widely considered. Previous research on Jersey cows revealed that by increasing the protein intake during the dry period, a lower number of milk fever incidences was observed (51). The authors proposed that circulating calcium concentration and metabolism might be affected by protein intake in dairy cows. Nevertheless, the information on the impact of individual AA feeding is scarce. de Moraes et al. (52) examined the impact of dietary Met concentration on performance, localization, and expression of the epithelial calcium transporter channels of digestive and reproductive systems in laying quails under heat stress. The authors pinpointed that the negative effect of heat stress was minimized when the birds took Met concentration at a 120% requirement level. In addition, epithelial calcium transporters were altered; hence, the animals required less calcium absorption and re-absorption due to more available calcium upon Met supplementation. Both Met and Zn are important factors for whole body protein synthesis, especially binding proteins (52). However, a combination of Zn and Met seems more effective. Therefore, a complex of Zn-Met may influence the blood calcium flux by increasing the synthesis of binding protein and optimizing the epithelium structure, which fascinate the absorptive and re-absorptive potential of calcium. Horst et al. (53) argued that changes in circulating calcium are simply reflective of animal homoerotic and immune activation. The authors mentioned hypocalcemia might occur as a reflection of inflammation and the health status of animals, which is associated with poor performance responses. It could be postulated that healthy cows under heat stress encompass a better metabolic pattern as well as suffer less from acute inflammation. Moreover, a positive immune system resulted in normal calcium metabolism, demonstrating a non-hypocalcemia status (53). The circulating Zn concentration of the animals fed the dietary Zn-Met complex was clearly higher. A consequence of an increased Zn absorption in the gastrointestinal tract would be a higher mineral concentration in blood as observed in the current study. Previous reports indicated that Zn bioavailability is increased in the dairy cows fed experimental diets with an organic form of Zn (19). This would lead to improved productive and metabolic responses in the animals (15, 43).

In dairy cows under energy deficiency, a mobilization of body fat reserves occurs, and thus, the concentration of NEFA increases in the blood (54). A non-significant difference in blood bioenergetics metabolites, as observed herein, could imply that the cows in both experimental groups were in relatively balanced energy status. Metabolic alteration in liver function is an important aspect in high-producing dairy cows, particularly during periods of heat stress. An elevation of SGPT and SGOT may indicate an accumulation of NEFA transported from blood to hepatocytes (55), which in turn enables the liver to reduce hepatocyte damage and moderate its metabolic function. Non-significant differences in the blood enzyme concentration observed in this experiment could suggest that the animals in both experimental groups had a healthy liver function. Nevertheless, previous work showed that liver enzyme activities in cattle exposed to thermal stress were evidently lower (56). In addition, holistic proteome analysis of liver tissues of heat-stressed dairy cattle revealed lower ATP synthesis and alteration in lipids, carbohydrates, and AA metabolism (57). A plausible reason for a less difference in animal liver metabolism in our work might be the duration of the study. In contrast to the current study, Skibiel et al. (57) determined the impact of heat stress in animals during the transition period, which is a time frame with more pressure on the metabolic activity of animals.

### Selected inflammatory and oxidative biomarkers

The severity of inflammation in dairy cattle can be characterized by an increased circulating concentration of haptoglobin, a positive acute phase protein, and lower biosynthesis of albumin, a negative acute phase protein (58). Haptoglobin levels were shown to be evidently higher in non-healthy dairy cows than in healthy animals (59). Previous studies indicated elevated levels of haptoglobin as early as 5-day post-partum in cows developing subclinical endometritis in later stages post-calving (60). These findings provide evidence that blood haptoglobin concentration, as a metabolic signal, is associated with systemic inflammatory responses and liver function (61). Lower haptoglobin levels in the RPZM group indicated that animals were less prone to experience acute inflammation, thereby being able to maintain their health status during the LEHS period. Heat stress alters the use of AAs in metabolic tissues by decreasing the blood AA concentration and increasing nitrogen excretion. This could limit the function of AA supply to the liver and mammary gland, resulting in an increased inflammatory response. Boosting the post-ruminal Met supply during heat stress might lead to a lower acute inflammation status, which in turn supports the overall health of the animals. Bertoni et al. (58) found that cows with high indexes of inflammation produced 20% less milk during the first month of lactation than those in the lower index category. Our results confirmed that dietary supplementation of high-producing dairy cows with the Zn-Met complex during LEHS would have a positive effect on the animal health status, ultimately improving the lactation performance of the animals. Lopreiato et al. (62) using an *in vitro* experiment showed that Met at the lowest concentrations had the potential to downregulate the entire repertoire of innate inflammatory genes, such as IL-1B and−6, proposing reduced cellular inflammation. IL-1 is present in either a secreted or membrane-bound form in many cell types, for example, hematopoietic and non-hematopoietic cells. The expression of IL-1 could be triggered by various stimuli, such as inflammation and stress, which ensures host protection and pathogen control by driving cell–cell cross-interaction (63). Mehla et al. (64) revealed that IL-1 gene expression increased during short exposure periods of heat stress while decreasing in the recovery phase. Previous works have shown higher levels of different cytokines, such as IL-1B, IL-6, and tumor necrosis factor-α, in heat stress-exposed cows, which suggests the possibility of an inflammatory condition resulting from the heat stress environment (65). Lower serum IL-1B levels upon Zn-Met complex supplementation in the current experiment indicated that the animals were able to adjust more efficiently to the potent negative impacts of thermal stress on the dairy cow immune system.

The lower MDA concentration in the dairy cows fed with the Met-Zn complex observed herein pinpoints a reduction in lipid peroxidation in these animals. Oxidative stress is an imbalance of antioxidants and oxidative molecules, such as reactive oxygen species and lipid peroxides. Malondialdehyde exerts cytotoxic effects since it oxidizes DNA, proteins, and lipids and establishes cross-links between AA side groups. Therefore, MDA detection implies systemic effects of persisting oxidative stress. Previous reports revealed that heat stress exacerbates oxidative stress and reactive oxygen species production in dairy cows (66). The results of this study were in line with previous works indicating lower levels of lipid peroxidation and improved antioxidant capacity in mid-lactating dairy cows fed Met derivatives (67). Increasing post-ruminal Met in ewes resulted in an evident decline in MDA plasma levels, as observed in the current experiment (68). The outcome of this experiment was in contrast to previous reports in which supplementing the diet of dairy cows with Zn-Met did not show any significant effect on blood MDA or TAS levels (69). Oxidative stress is one of the contributing factors to the impairment of the immune responses during environmental stress. Hence, alteration of oxidative metabolites in cows would influence cytokine production (70). The outcome of the current experiment indicates that dairy cows benefit from feeding a higher bioavailable source of Zn and Met by reactivating their immune system and expressing an adaptive response.

## Conclusion

Overall, a rumen-protected Zn-Met complex dietary inclusion ameliorates the production performance of dairy cows during the period of LEHS. The feed intake of the animals was not influenced by the dietary inclusion of the rumen-protected Zn-Met. Nonetheless, milk production corrected for both fat and energy was higher upon the Zn-Met complex supplementation. Milk composition, particularly milk fat and protein concentration, in the animals supplemented with the rumen-protected Zn-Met complex was improved in the current experiment. Higher productivity in the animals seems to be partly driven by lower inflammation and improved antioxidant capacity, as observed by the selected blood biomarkers. Environmental heat stress can initiate oxidative stress in animals, deteriorating their productive responses and health status. The Zn-Met complex seems to alleviate those potent negative impacts of oxidative stress. In addition, lower haptoglobin levels along with decreased IL-1B serum concentration in the rumen-protected Zn-Met-supplemented dairy cattle suggest that the animals would suffer less from acute or chronic diseases, allowing them to maintain or even improve their productive capacity during LEHS. Elaboration of the detailed mechanism merits further investigation. It is compelling to determine the influence of the rumen-protected Zn-Met source during transition period, where dairy cows liver function and immune system is greatly challenged.

## Data availability statement

The original contributions presented in the study are included in the article/Supplementary material, further inquiries can be directed to the corresponding author/s.

## Ethics statement

The animal study was reviewed and approved by Institutional Animal Care Committee, Ferdowsi University of Mashhad (Mashhad, Iran; Protocol number 101984).

## Author contributions

MDM, HK, SDM, and RJ conceived the project. MDM and HK designed the study and wrote the paper with a critical reviewed by all authors. HK conducted the experiment and sample collection. AG helped with parts of the sample analysis and conducted the statistical analysis. AV performed part of the blood analysis. All authors reviewed and approved the final manuscript.

## Funding

Financial support for this experiment was provided in part by Ferdowsi University of Mashhad (Grant Number: 9305) and Kaesler Nutrition GmbH (Cuxhaven, Germany).

## Conflict of interest

Authors RJ and SDM were employed by Kaesler Nutrition GmbH. The remaining authors declare that the research was conducted in the absence of any commercial or financial relationships that could be construed as a potential conflict of interest.

## Publisher's note

All claims expressed in this article are solely those of the authors and do not necessarily represent those of their affiliated organizations, or those of the publisher, the editors and the reviewers. Any product that may be evaluated in this article, or claim that may be made by its manufacturer, is not guaranteed or endorsed by the publisher.
