# Supplementary material for: Rumen-protected zinc–methionine dietary inclusion alters dairy cow performances, and oxidative and inflammatory status under long-term environmental heat stress
Source: Front Vet Sci. 2022 Sep 12;9:935939. doi: 10.3389/fvets.2022.935939 (PMC9510689; doi:10.3389/fvets.2022.935939)
Supplement: Supplementary file 1 [file Data_Sheet_1.docx]

Supplementary Material

**Supplementary Table S1.** Ingredients and chemical composition of the experimental diets fed to the dairy cows

| Items | Control^1^ | RPZM^2^ |
| --- | --- | --- |
| **Ingredient, % of DM^3^** |  |  |
| Corn silage | 21.11 | 21.11 |
| Alfalfa hay | 20.31 | 20.31 |
| Wheat Straw | 0.40 | 0.40 |
| Corn grain | 13.32 | 12.32 |
| Barley grain | 12.58 | 12.58 |
| Sugar beet pulp | 1.80 | 1.80 |
| Extruded soybean meal | 10.48 | 10.48 |
| Wheat bran | 3.52 | 3.52 |
| Cottonseed whole | 6.30 | 6.30 |
| Cottonseed meal | 2.80 | 2.80 |
| Rape seed meal | 3.90 | 3.77 |
| Supplement^4^ | 3.50 | 3.50 |
| Loprotin | 0.0 | 0.13 |
| Chemical composition |  |  |
| CP^5^, g/kg DM  Rumen CP degradability, g/kg DM | 166  118 | 166  118 |
| NDF^6^, g/kg DM | 346 | 346 |
| ADF^7^, g/kg DM | 178 | 178 |
| Ash, g/kg DM | 51 | 51 |
| Starch+soluble surges, g/kg DM | 296 | 296 |
| EE^8^, g/kg DM | 47 | 47 |
| Zn, g/kg DM | 0.03 | 0.12 |
| ME^9^, Mj/kg DM | 11.9 | 11.9 |
|  |  |  |

^1^Control= Experimental diet without rumen-protected Zn-Met supplementation

^2^RPZM= Experimental diet with rumen-protected Zn-Met supplementation as 0.13% of diet dry matter

^3^dry matter

^4^Containing (/kg dry matter) 180 g Sodium bicarbonate (Petrotarh, Iran), 60 g Di-calcium phosphate (www.javanehkhorasan.com), 200 g VitalG (Rumen protected glucose, www.groupsana.comena), 150 g Optimate (Essential omega-3 from Salmen oil, rumen protected with vitamins, www.agritech.ie), 25 g Lutrell® Pure (Conjugated Linoleic Acid (CLA), BASF), 30 g encapsulated Choline chloride (www.Kemin.com), 60 g vitamin D3 (5000 000 IU/kg), 80 g vitamin E & Se (11000 IU & 300 mg/kg, respectively), 180 g mineral/vitamin premix (vitamins including A: 1500000 IU, D3:400000 IU, E:3000 IU, Biotin: 120 mg; minerals including Ca, P, Mg Na, K, Mn, Cu, Se, I, Fe, Co and S with the quantity of 44 g , 20 g, 2.3 g, 20 g, 1.6 g, 3.4 g, 0.14 g, 0.25 g, 0.043 g and 17.6 g, respectively, www.javanehkhorasan.com), 35 g Toxin bonder (Toxytrap, Iran).

^5^Crude protein

^6^Neutral detergent fiber

^7^Acid detergent fiber

^8^Ether extract

^9^Metabolizable energy

**Supplementary Table S2.** Dry matter intake and milk performance of high producing Holstein cows supplemented without or with rumen-protected Zinc-Methionine complex during the long-term environmental heat stress. *When the difference between means is greater than two times the standard error of means (SEM), it is considered as significant (P< 0.05).

|  |  | Experimental groups* | | | | | | | | | | | | SEM | p-value | | | |
| --- | --- | --- | --- | --- | --- | --- | --- | --- | --- | --- | --- | --- | --- | --- | --- | --- | --- | --- |
|  | Week of lactation  (Control^1^) | | | | | |  | Week of lactation  (RPZM^2^) | | | | | |  | Groups | Week | group×week | |
|  | W6 | W7 | W8 | W9 | W10 | W11 |  | W6 | W7 | W8 | W9 | W10 | W11 |  |  |  |  |  |
| DMI^3^, kg/d | 25.3 | 26.2 | 25.9 | 26.4 | 25.7 | 25.6 |  | 25.9 | 26.8 | 27.1 | 26.2 | 26.9 | 27.2 | 1.3 | 0.410 | 0.761 | 0.811 |  |
| Milk production, kg/d | 51.2 | 51.7 | 53.1 | 53.0 | 55.1 | 53.5 |  | 53.6 | 55.2 | 55.4 | 55.5 | 55.7 | 54.7 | 1.21 | 0.300 | 0.032 | 0.647 |  |
| Milk composition g/kg |  |  |  |  |  |  |  |  |  |  |  |  |  |  |  |  |  |  |
| Fat | 31.8 | 27.3 | 24.0 | 32.4 | 21.7 | 26.5 |  | 32.4 | 35.2 | 28.1 | 34.8 | 32.8 | 33.5 | 1.82 | <.01 | <.01 | 0.031 |  |
| Protein | 30.7 | 29.6 | 30.4 | 30.2 | 29.8 | 30.1 |  | 31.0 | 30.6 | 31.3 | 31.4 | 30.4 | 31.5 | 0.23 | <.01 | <.01 | 0.219 |  |
| Lactose | 46.2 | 46.4 | 46.9 | 45.8 | 47.0 | 47.2 |  | 46.0 | 45.3 | 46.6 | 45.4 | 46.1 | 45.7 | 0.33 | 0.008 | <.01 | 0.284 |  |
| Solids | 118 | 113 | 111 | 118 | 109 | 114 |  | 120 | 121 | 116 | 121 | 119 | 121 | 1.75 | <.01 | <.01 | 0.044 |  |
| SNF^4^ | 86.8 | 85.9 | 87.4 | 85.9 | 86.9 | 87.1 |  | 87.0 | 85.7 | 88.0 | 86.2 | 86.3 | 87.2 | 0.45 | 0.456 | <.01 | 0.815 |  |
| MUN^5^, mg/dl | 13.5 | 13.8 | 13.9 | 13.5 | 16.3 | 13.8 |  | 14.1 | 14.2 | 14.0 | 13.5 | 15.4 | 14.0 | 4.87 | 0.683 | <.01 | 0.730 |  |
| Milk composition yield, kg/d |  |  |  |  |  |  |  |  |  |  |  |  |  |  |  |  |  |  |
| Fat | 1.6 | 1.4 | 1.2 | 1.7 | 1.2 | 1.4 |  | 1.7 | 1.9 | 1.5 | 1.9 | 1.8 | 1.8 | 0.10 | <.01 | <.01 | 0.063 |  |
| Protein | 1.57 | 1.53 | 1.62 | 1.6 | 1.64 | 1.61 |  | 1.67 | 1.68 | 1.74 | 1.74 | 1.69 | 1.72 | 0.04 | 0.01 | 0.10 | 0.605 |  |
| Lactose | 2.37 | 2.41 | 2.5 | 2.44 | 2.59 | 2.52 |  | 2.47 | 2.5 | 2.58 | 2.51 | 2.57 | 2.5 | 0.06 | 0.675 | 0.005 | 0.521 |  |
| Solids | 6.0 | 5.8 | 5.9 | 6.3 | 6.0 | 6.1 |  | 6.4 | 6.7 | 6.4 | 6.7 | 6.6 | 6.6 | 0.17 | 0.042 | 0.198 | 0.528 |  |
| SNF | 4.45 | 4.45 | 4.64 | 4.55 | 4.79 | 4.66 |  | 4.67 | 4.73 | 4.87 | 4.77 | 4.8 | 4.77 | 0.10 | 0.234 | 0.024 | 0.616 |  |
| 4% FCM^6^, kg/d | 44.4 | 41.4 | 39.6 | 46.9 | 40.1 | 42.6 |  | 47.0 | 51.0 | 45.3 | 50.9 | 49.5 | 49.1 | 1.82 | 0.002 | 0.003 | 0.140 |  |
| ECM^7^, kg/d | 48.2 | 45.3 | 44.4 | 50.5 | 45.0 | 46.9 |  | 51.1 | 54.6 | 50.1 | 54.8 | 53.3 | 53.2 | 1.72 | 0.004 | 0.009 | 0.211 |  |
| NE_L_^8^, Mcal/d | 92.9 | 88.0 | 91.1 | 95.0 | 87.0 | 92.8 |  | 98.0 | 100 | 100 | 104 | 96.9 | 102 | 2.71 | 0.024 | 0.010 | 0.743 |  |
| SCC^9^, x1000/ml | - | 85.9 | - | 209 | - | 157 |  | - | 51.5 | - | 67.5 |  | 19.3 | 10.40 | 0.001 | 0.121 | 0.561 |  |

^1^Control= Experimental diet without rumen-protected Zn-Met supplementation

^2^RPZM= Experimental diet with rumen-protected Zn-Met supplementation as 0.13% of diet dry matter

^3^DMI= Dry matter intake

^4^SNF= Solids nonfat

^5^MUN= Milk urea nitrogen

^6^FCM= Fat corrected milk; FCM = [0.4 × milk yield (kg)] + [15 × milk fat (kg)]

^7^ECM= Energy corrected milk; ECM= [0.3246 × milk yield (kg)] + [12.86 × fat yield (kg)] + [7.04 × protein yield (kg)]

^8^NEL= NEL= [(0.0929 × % milk fat) + (0.0563 × % milk true protein/0.93) + (0.0395 × % milk lactose)] × milk yield

^9^SCC= somatic cell scores

**Supplementary Table S3.** Concentration of blood serum metabolites of high producing Holstein cows, through lactation week 6 to 11, supplemented without or with rumen-protected Zinc-Methionine complex during the long-term environmental heat stress. *When the difference between means is greater than two times the standard error of means (SEM), it is considered as significant (P< 0.05).

|  |  | | Experimental groups | | | | | | | |  | p-value | | |
| --- | --- | --- | --- | --- | --- | --- | --- | --- | --- | --- | --- | --- | --- | --- |
| Items | Week of lactation | | | | | |  | Week of lactation | | | SEM | Groups | Week | group×week |
|  | (Control^1^) | | | | | |  | (RPZM^2^) | | |  |  |  |  |
| Items | W7 | | W9 | | W11 | |  | W7 | W9 | W11 |  |  |  |  |
| Glucose mg/dL | 37.2 | | 42.6 | | 44.3 | |  | 36.5 | 42.4 | 42.1 | 1.66 | 0.521 | 0.001 | 0.788 |
| Urea, mg/dL | 53.5 | | 54.5 | | 55.8 | |  | 53.9 | 54.5 | 57.5 | 1.94 | 0.765 | 0.107 | 0.823 |
| Cholesterol, mg/dL | 179 | | 191 | | 201 | |  | 190 | 224 | 225 | 10.78 | 0.100 | <.01 | 0.208 |
| HDL^3^, mg/dl | 88.1 | | 90.9 | | 89.1 | |  | 89.1 | 98.6 | 103 | 5.72 | 0.270 | 0.099 | 0.197 |
| LDL^4^, mg/dL | 87.0 | | 96.7 | | 108 | |  | 97.4 | 122 | 119 | 7.09 | 0.061 | 0.004 | 0.269 |
| Triglyceride, mg/dL | 20.2 | | 18.1 | | 17.0 | |  | 19.4 | 17.5 | 16.3 | 1.18 | 0.536 | 0.018 | 0.992 |
| VLDL^5^, mg/dL | 4.04 | | 3.61 | | 3.40 | |  | 3.88 | 3.51 | 3.25 | 0.24 | 0.536 | 0.018 | 0.992 |
| SGOT^6^, U/L | 147 | | 147 | | 151 | |  | 127 | 132 | 139 | 8.82 | 0.162 | 0.277 | 0.782 |
| SGPT^7^, U/L | 36.2 | | 35.8 | | 36.5 | |  | 34.9 | 41.9 | 38.1 | 2.64 | 0.431 | 0.341 | 0.255 |
| Protein, g/dL | 8.71 | | 9.22 | | 8.71 | |  | 8.78 | 9.65 | 9.06 | 0.18 | 0.137 | <.01 | 0.441 |
| Albumin, g/dL | 4.28 | | 4.37 | | 4.21 | |  | 4.25 | 4.61 | 4.44 | 0.08 | 0.080 | 0.001 | 0.038 |
| Globulin, g/dL | 4.43 | | 4.85 | | 4.50 | |  | 4.53 | 5.03 | 4.62 | 0.19 | 0.552 | 0.003 | 0.932 |
| A/G^8^ | 1.00 | | 0.94 | | 0.97 | |  | 0.95 | 0.92 | 0.97 | 0.04 | 0.645 | 0.096 | 0.458 |
| Calcium, mg/dL | 9.88 | | 10.6 | | 10.6 | |  | 10.8 | 11.9 | 11.3 | 0.23 | <.01 | 0.002 | 0.460 |
| Zinc, ug/dL | 80.7 | | 75.4 | | 67.1 | |  | 95.3 | 86.1 | 77.9 | 5.36 | 0.038 | 0.005 | 0.887 |
| NEFA^9^, mmol/L | 0.47 | | 0.51 | | 0.40 | |  | 0.44 | 0.56 | 0.33 | 0.06 | 0.771 | 0.006 | 0.442 |
| BHB^10^, mmol/L | 0.68 | | 0.66 | | 0.61 | |  | 0.68 | 0.65 | 0.59 | 0.04 | 0.783 | 0.084 | 0.923 |
| TAS^11^, mmol/L | 0.63 | | 0.61 | | 0.63 | |  | 0.68 | 0.69 | 0.68 | 0.02 | 0.017 | 0.915 | 0.718 |
| MDA^12^, nmol/mL | 2.70 | | 1.65 | | 1.69 | |  | 2.11 | 1.61 | 1.57 | 0.15 | 0.082 | <.01 | 0.119 |
| Haptoglobin, mcg/mL | 269 | | 268 | | 264 | |  | 247 | 232 | 240 | 10.53 | 0.001 | 0.604 | 0.665 |
| IL-1B^13^, ng/L | 78.7 | 73.0 | | 78.7 | | 54.9 | | | 56.6 | 68.6 | 3.4 | 0.001 | 0.001 | 0.22 |

^1^Control= Experimental diet without rumen-protected Zn-Met supplementation

^2^RPZM= Experimental diet with rumen-protected Zn-Met supplementation as 0.13% of diet dry matter

^3^HDL= High-density lipoprotein

^4^LDL= Low-density lipoprotein

^5^VLDL= Very-low-density lipoprotein

^6^SGOT= Aspartate aminotransferase

^7^SGPT= Alanine aminotransferase

^8^A/G= Albumin to globulin ratio

^9^NEFA= Non-esterified fatty acids

^10^BHB= Beta-hydroxybutyrate

^11^TAS= Total antioxidant status

^12^MDA= Malondialdehyde

^13^IL-1B= Interleukin-1 beta
